# Maternity, Infancy and Childhood

**Published:** 1887-06

**Authors:** 


**Maternity, Infancy and Childhood.** Hygiene of Pregnancy, Nursing and Weaning of Infants; the Care of Children in Health and Disease. By John M. Keating, Lecturer on Diseases of Women and Children, Philadelphia Hospital. J. B. Lippincott & Co., Philadelphia. London, 10 Henrietta st., Covent Garden.

This is the second volume of the excellent series of "Practical Lessons in Nursing," now being issued by the Lippincotts at the low price of one dollar each. It is specially intended and adapted for the use of mothers and those intrusted with the bringing up of children, and it is a book which the practitioner can safely commend to his families. The chapters on infant feeding and the weaning of children can be read by most physicians with interest and profit.

